# *Lactobacillus crispatus* BC1 Biosurfactant Delivered by Hyalurosomes: An Advanced Strategy to Counteract *Candida* Biofilm

**DOI:** 10.3390/antibiotics10010033

**Published:** 2021-01-01

**Authors:** Angela Abruzzo, Barbara Giordani, Carola Parolin, Priscilla R. De Gregorio, Claudio Foschi, Teresa Cerchiara, Federica Bigucci, Beatrice Vitali, Barbara Luppi

**Affiliations:** 1 Department of Pharmacy and Biotechnology, Alma Mater Studiorum—University of Bologna, Via San Donato 19/2, 40127 Bologna, Italy; angela.abruzzo2@unibo.it (A.A.); barbara.giordani4@unibo.it (B.G.); teresa.cerchiara2@unibo.it (T.C.); federica.bigucci@unibo.it (F.B.); b.vitali@unibo.it (B.V.); barbara.luppi@unibo.it (B.L.); 2 Centro de Referencia para Lactobacilos (CERELA)-CONICET, Chacabuco 145, 4000 San Miguel de Tucumán, Argentina; pridegregorio@cerela.org.ar; 3 Department of Experimental, Diagnostic and Specialty Medicine, Alma Mater Studiorum—University of Bologna, Via Massarenti 9, 40138 Bologna, Italy; claudio.foschi2@unibo.it

**Keywords:** biosurfactant, *Lactobacillus*, liposomes, hyalurosomes, mucoadhesion, *Candida*, biofilm

## Abstract

The emergence of resistance to antifungal drugs has made the treatment of vulvovaginal candidiasis (VVC) very challenging. Among natural substances, biosurfactants (BS) produced by *Lactobacillus* have gained increasing interest in counteracting *Candida* infections for their proven anti-adhesive properties and safety profile. In the present study, liposomes (LP-BS) or liposomes coated with hyaluronic acid (HY-LP-BS) were prepared in the presence of the BS isolated from the vaginal strain *Lactobacillus crispatus* BC1 and characterized in terms of size, ζ potential, stability and mucoadhesion. The anti-biofilm activity of free BS, LP-BS and HY-LP-BS was investigated against different *Candida albicans* and non-*albicans* strains (*C. glabrata*, *C. lusitaniae*, *C. tropicalis*, *C. krusei* and *C. parapsilosis*), clinically isolated from patients affected by VVC. The inhibition of biofilm formation and the dispersal of pre-formed biofilm were evaluated. The obtained phospholipid vesicles showed suitable size for vaginal application and good stability over the storage period. HY-LP-BS exhibited good mucoadhesive properties and the best anti-biofilm profile, both in preventing or limiting the surface colonization by a broad spectrum of *Candida* species. In conclusion, the formulation of a novel antifungal agent derived from the vaginal microbiota into mucoadhesive nanocarriers appears to be a promising biotherapeutic strategy to counteract vulvovaginal candidiasis.

## 1. Introduction

Vulvovaginal candidiasis (VVC) is a multifactorial infectious disease of the lower female reproductive tract and it represents one of the most common vaginal infections worldwide. More than 90% of VVC cases are caused by *Candida albicans*, but non-*albicans Candida* species, such as *C. glabrata*, *C*. *krusei*, *C*. *parapsilosis*, and *C*. *tropicalis*, have been also identified as etiological agents. While the epidemiology of infection varies among these species, their general virulence attributes appear mostly conserved. Concerning virulence, a majority of invasive infections are related to the microbial ability to live and grow in a sessile form. Biofilms are closely packed communities of microbial cells that adhere to surfaces, such as tissues and implanted medical devices. *Candida* species are commonly able to produce biofilms and acquire drug resistance; drug sequestration by the biofilm matrix is responsible for much of the antifungal tolerance. Consequently, infections involving biofilm establishment are challenging to cure [1,2]. Considering the high incidence and recurrence of VCC and the development of intrinsically resistant *Candida* species as a consequence of extensive use of antifungal azoles [3,4], new treatment strategies are extremely desirable. Biosurfactants (BS), surface active agents produced by living microorganisms as secondary metabolites [5], have been proven to exert anti-microbial and anti-biofilm activity against different human pathogens and can represent potential candidates for the treatment of local infections [6]. In particular, biosurfactants isolated from probiotic bacteria, including *Lactobacillus*, may offer the opportunity to deliver health benefits, as probiotics are part of healthy microbiota and therefore are beneficial for humans and safe for therapeutic purposes [7,8]. In a recent study, a BS isolated from vaginal *Lactobacillus crispatus* BC1 was found to interfere with the adhesion of various *Candida* isolates to human cervical epithelial cells; furthermore, BS intravaginal inoculation in a murine experimental model was shown to be safe and effective in reducing leukocyte influx in the case of *C*. *albicans* infection [9]. *L. crispatus* species plays a crucial role in maintaining vaginal eubiosis, exerting beneficial properties that have already been extensively addressed in previous studies [10,11,12,13,14]. Therefore, the employment of metabolites or derivatives from this species as therapeutic agents represents an interesting and stimulating challenge in the field of women’s health maintenance.

Together with the use of alternative anti-*Candida* agents based on vaginal probiotics, the development of suitable carriers could further improve the treatment. In recent years, liposomes have demonstrated their potential to counteract local infections due to their ability to guarantee a modified release of the active substance. Such a property ensures an adequate concentration of the active drug at the administration site [15,16]. At the same time, different scientists have shown that liposomal vesicles could prevent the formation as well as favor the eradication of microbial biofilms [17], including those formed by bacteria resistant to antibiotics [18]. Extended local treatment could be favored by appropriate residence time of the carrier at the application site; thus, coating materials based on mucoadhesive polymers represent a further option to maximize the delivering ability of phospholipid vesicles. Hyaluronic acid has been used as a mucoadhesive agent for the coating of liposomal vesicles [19] and also as a hydrating and healing agent for the treatment of vulvo-vaginal atrophy [20]. Moreover, high molecular weight hyaluronic acid was found to be involved in the physical enhancement of the epithelial barrier and the improvement of its innate immune response, thus suggesting it may protect the urogenital tract from microbial infections [21]. Due to these interesting functional properties, it can represent the right option for the development of a vaginal delivery system intended for the treatment of VVC.

In the present work, two novel strategies have been merged: the choice of a new potential antifungal agent derived from the human healthy microbiota and the use of mucoadhesive nanocarriers for its vaginal delivery. Two different types of liposomes, prepared in the presence of the BS isolated from the vaginal strain *Lactobacillus crispatus* BC1, were evaluated for their ability to inhibit biofilm formation and disperse pre-formed biofilm of different *C. albicans* and non-*albicans* clinically isolated strains.

## 2. Results and Discussion

In a previous work, we demonstrated the ability of BS produced by the vaginal strain *L. crispatus* BC1 (BC1-BS) to interfere with the adhesion of *Candida* spp. to HeLa cells. Pathogen adhesion represents the first step in the mucosal colonization and the subsequent biofilm development. BC1-BS was chemically characterized and found to contain an amino acid portion attributable to tyrosine, serine, proline, glycine and arginine linked to fatty acids, namely β-hydroxytridecanoic acid (3-OH-C13), β-hydroxytetradecanoic acid (3-OH-C14), β-hydroxypentadecanoic acid (3-OH-C15) and β-hydroxyhexadecanoic acid (3-OH-C16). Moreover, BC1-BS is able to reduce the surface tension and it showed a critical micellar concentration equal to 2 mg/mL. BC1-BS revealed a good anti-adhesion activity tested against clinically relevant *Candida* spp. for concentrations up to 1.25 mg/mL, which was also found not to be cytotoxic to HeLa cells [9]. Thus, considering a possible vaginal application, in the present study we formulated liposomes and hyalurosomes at a final BS concentration of 1.25 mg/mL.

### 2.1. Determination of Vesicle Size Distribution and ζ Potential

When nanocarriers are intended for local vaginal application, their size and surface charge play an important role in improving the treatment. In the present study, conventional liposomes (LP) or liposomes coated with hyaluronic acid (i.e., hyalurosomes, HY-LP) were prepared; phospholipid vesicles containing BC1-BS were developed, giving rise to LP-BS and HY-LP-BS. The main physicochemical properties of the developed vesicles are summarized in Table 1. HY-LP showed a greater size with respect to LP (*p* < 0.05), probably as a consequence of polymer adsorption on the lipid bilayer. This result is in agreement with our recent findings [22] and previous observations by other authors [23]. Specifically, it was reported that hyaluronic acid could be adsorbed and consequently intercalated into the lipid bilayers, thus leading to an increase in phospholipid vesicle size [22,23].

On the other hand, for both liposomes and hyalurosomes, the presence of BC1-BS (LP-BS and HY-LP-BS) led to the formation of smaller structures with respect to the phospholipid vesicles prepared without biosurfactant (LP and HY-LP, respectively) (*p* < 0.05). This behavior is probably correlated with the particular composition of the biosurfactant. In fact, as reported in De Gregorio et al., BC1-BS was characterized by the presence of different fatty acids and amino acids, including arginine [9]. The positive charges on arginine residues could reduce the repulsive forces between the bilayers and consequently decrease the vesicle size. This observation conforms to previous results. In particular, the addition of cationic molecules, such as cationic surfactants in liposomal formulation, was found to decrease phospholipid vesicle size due to a reduction in the repulsive forces between the lipidic bilayers [24,25,26]. Moreover, the lipophilic portion could contribute to the decrease in size in agreement with other results. Duangjit and co-workers [25] investigated the impact of carbon chain length and content of different surfactants on meloxicam-loaded liposomes. They obtained a reduction in size with the increase of the length of surfactant carbon chain and attributed this result to the improvement of the solubility of the surfactant molecules within the lipid bilayer and to the consequent increase in vesicle rigidity. Additionally, the lipophilic portion of the surfactant exhibited strong hydrophobic interactions with phosphatidylcholine, thus determining the formation of a tighter vesicle bilayer [25,27]. However, taking into account that nanocarriers in the size range of 200–500 nm can deliver molecules to vaginal tissue more efficiently than both smaller and larger carriers [28], all the prepared phospholipid vesicles could be suitable for vaginal application and BS delivery.

Polydispersity index value (PDI) is a measure of the width of unimodal size distributions [29]. In our study, PDI values for all the phospholipid vesicles were in general small, around 0.3, demonstrating a good dispersion homogeneity [30]. Indeed, this value is in agreement with other observations reporting that a PDI of 0.3 or below is considered to be acceptable and indicates a homogenous population of phospholipid vesicles [31,32,33,34,35].

Phospholipid vesicles were characterized by negative ζ potential values, as a consequence of the presence of phosphatidylcholine phosphate groups that in saline solution (pH = 6.0) were negatively charged, in agreement with other published works [18,22,36,37]. Despite the polyanionic nature of the polymer and its adsorption on the lipidic bilayer, no significant difference was observed between ζ potential values of HY-LP and LP (*p* > 0.05), probably due to the low polymer concentration (0.01% *w/v*). This datum is in agreement with that of a previous work, in which similar results were obtained when 0.2% w/v polymer concentration was used [23]. Finally, the presence of BS in liposomes and hyalurosomes led to an increase in ζ potential (*p* < 0.05). This result can be attributable to the presence of positively charged residues of arginine on the BS structure that could interact with the anionic phosphate groups of phosphatidylcholine, providing a more positive structure. Our data agree with the findings of Duangjit and co-workers, who observed an increase of positive charges on the liposome surface when it contained a cationic surfactant [25].

### 2.2. Vesicle Physical Stability

**Figure 1:**
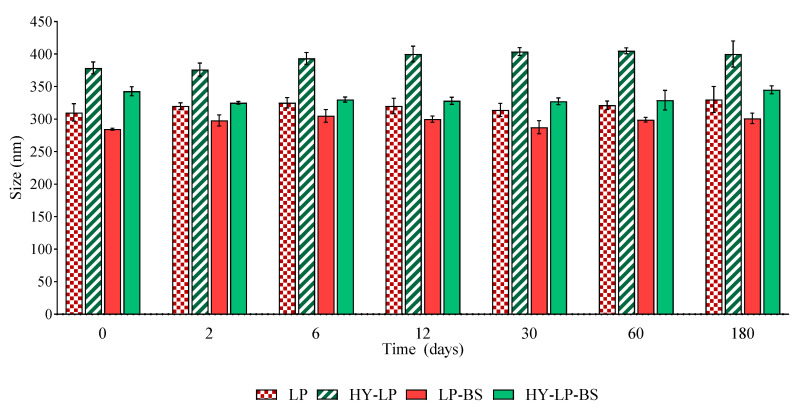
Variation of size of liposomes (LP) and hyalurosomes (HY-LP) with and without biosurfactants (BS) during 180 days of storage at 4–8 °C (mean ± SD, *n* = 3).

The effect of storage on the main properties of phospholipid vesicles was assessed by monitoring the changes of size and PDI over a storage period of 180 days at 4–8 °C. The vesicle variation in terms of size is reported in Figure 1. No sedimentation was found in any samples immediately after their preparation. Furthermore, the size of all the vesicles remained constant during the storage period, excluding any phenomenon of aggregation and precipitation. No significant changes in PDI values were observed after 180 days of storage, thus confirming the homogeneous size distribution over the tested period (data not shown).

### 2.3. Mucoadhesive Properties

**Figure 2:**
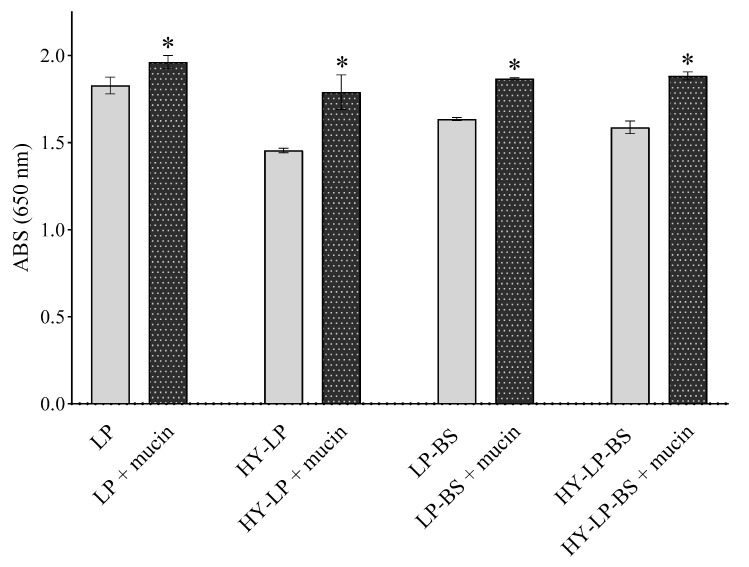
Absorbance at 650 nm measured for formulations incubated or not in the presence of mucin (mean ± SD, *n* = 3). The statistical significance with respect to formulation in absence of mucin was reported, *: *p* < 0.05.

A prerequisite for successful topical vaginal therapy is the formulation’s ability to guarantee a prolonged contact with the vaginal mucosa [38]. For this reason, mucoadhesive properties were investigated by measuring the turbidity at 650 nm of vesicle suspensions in the presence of mucin [16,39,40]. An increased turbidity with respect to the control (vesicle suspension without mucin) implies a greater mucoadhesion. For all the formulations, an increase of ABS was observed with respect to controls (Figure 2, *p* < 0.05). HY-LP showed a greater interaction with mucin compared to LP (% ABS increase 14.7 ± 1.0% and 7.4 ± 0.8%, respectively); probably as a consequence of the interaction between the polymer and the mucin chains. As reported in several studies, hyaluronic acid can interact with mucin in virtue of the chain entanglement and physical interlock with mucus and the presence of many hydrophilic groups that can establish hydrogen bonds [41]. Moreover, LP-BS and HY-LP-BS showed the highest mucoadhesive ability (% ABS increase 22.9 ± 5.6% and 18.6 ± 1.8%, respectively) among all the prepared phospholipid vesicles (*p* < 0.05), even if no significant difference was observed between liposomes and hyalurosomes (*p* > 0.05). The improvement of mucoadhesion could be ascribed to the presence of different aminoacids in BS structure able to interact with mucin. Specifically, as described before, the positively charged residues of arginine could interact with the negatively charged sialic acid (pKa = 2.6) and sulfate residues of mucin.

### 2.4. Evaluation of Anti-Biofilm Activity of Free BS, Liposomes and Hyalurosomes

Since the formation of *C. albicans* biofilm in vitro has been correlated with in vivo and ex-vivo models [42], in the present study we tested the anti-biofilm potential of BC1-BS by using 96 multi-well plates as abiotic surface. In particular, the anti-biofilm activity of free BC1-BS, LP-BS and HY-LP-BS was investigated against four *C. albicans* (*C. albicans* SO1-SO4) and five non-*albicans* (*C. glabrata* SO17, *C. lusitaniae* SO22, *C. tropicalis* SO24, *C. krusei* SO26 and *C. parapsilosis* SO27) strains, clinically isolated from patients affected by VVC. Moreover, two different experiments, namely the inhibition of biofilm formation and the dispersal of pre-formed biofilm, were taken into account.

#### 2.4.1. Inhibition of the Biofilm Development

**Figure 3:**
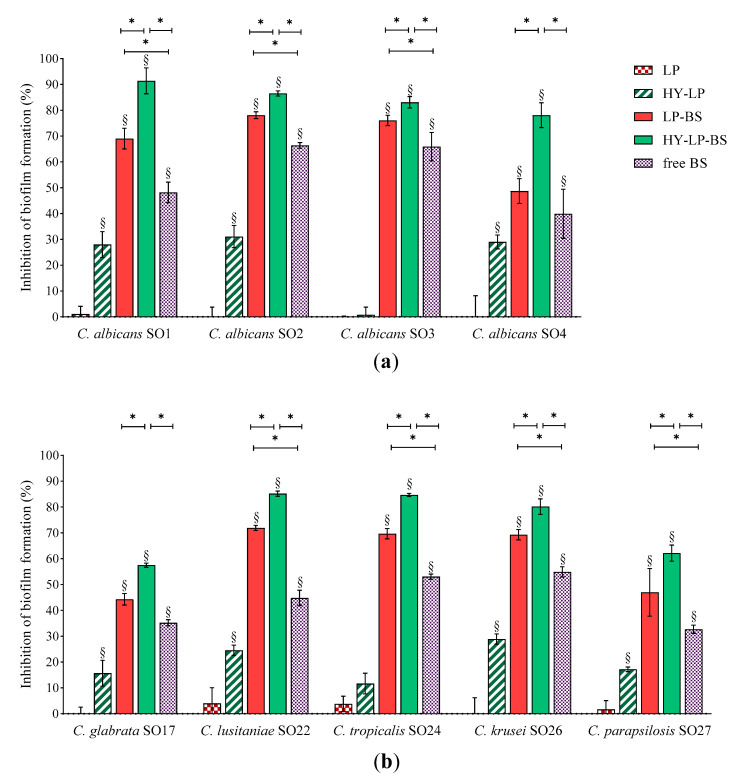
Anti-biofilm activity: inhibition of the development of (**a**) *C. albicans* and (**b**) non-*albicans* biofilms. Results are expressed as inhibition percentage (mean ± SD, *n* = 5). The statistical significance with respect to untreated control was reported, §: *p* < 0.01. Statistical differences between BS, LP-BS and HY-LP-BS were also calculated, *: *p* < 0.05.

For the inhibition assay, *Candida* suspensions were simultaneously incubated with free BC1-BS, LP-BS and HY-LP-BS and the biofilms were allowed to develop for 72 h, which is the average time required for biofilm maturation [43]. Results, expressed as inhibition percentages with respect to untreated control, are reported in Figure 3. Various studies have shown that biosurfactants can decrease the adhesion of microbial cells and colonization [44,45]. In this contest, free BS revealed a moderate ability to reduce the biofilm formation of all tested *Candida* strains with inhibition rates ranging from 44% to 66% for *C. albicans* strains (Figure 3a), and from 33% to 56% for non-*albicans* strains (Figure 3b). Similar results were reported by dos Santos et al. [46] for BS from *L. gasseri*, *L. paracasei* and *L. acidophilus* strains, even if results greatly varied depending on the origin of BS and *Candida* isolates.

The presence of BS in conventional liposomes (LP-BS) significantly enhanced their ability to interfere with the biofilm formation of seven *Candida* strains out of nine, while plain liposomes (LP) showed no activity. This result is in agreement with a previous study, which demonstrated the capability of liposomes to favor the anti-biofilm activity against *Staphylococcus aureus* of BS isolated from another *Lactobacillus* vaginal strain [18]. These findings suggest that phospholipid vesicles are a suitable nanocarrier to improve the biological effect of BC1-BS, probably favoring the interaction of active molecules with fungal surface [17].

In order to further ameliorate the anti-biofilm profile, a coating of hyaluronic acid was added to the formulation (HY-LP-BS). Interestingly, in this case the carrier itself (HY-LP) slightly reduced (*p* < 0.05) the biofilm formation of three *C. albicans* strains (inhibition rate of ~30%) and four non-*albicans* strains (inhibition rate of 15–28%), with the only exception being *C. tropicalis* SO24. Hyaluronic acid is known to possess antimicrobial activity against *Candida* [47,48], which can possibly impact also on fungal adhesion to the surface. Notably, the best anti-biofilm profile was observed for HY-LP-BS, which was significantly more active compared to free BS and LP-BS in counteracting the development of all *Candida* isolate biofilm, probably due to the combined contribution of BS and HY. In particular, HY-LP-BS strongly impaired the formation of biofilms by all tested *C. albicans* strains, with inhibition rates above 78%. The impact on non-*albicans* strains was slightly lower, with inhibition percentages ranging from 57% to 85%. These results suggest that the proposed vaginal formulation can be effective in preventing or limiting the surface colonization by a broad spectrum of *Candida* species, and thus reducing the severity of infection.

#### 2.4.2. Dispersal of Pre-Formed Biofilm

**Figure 4:**
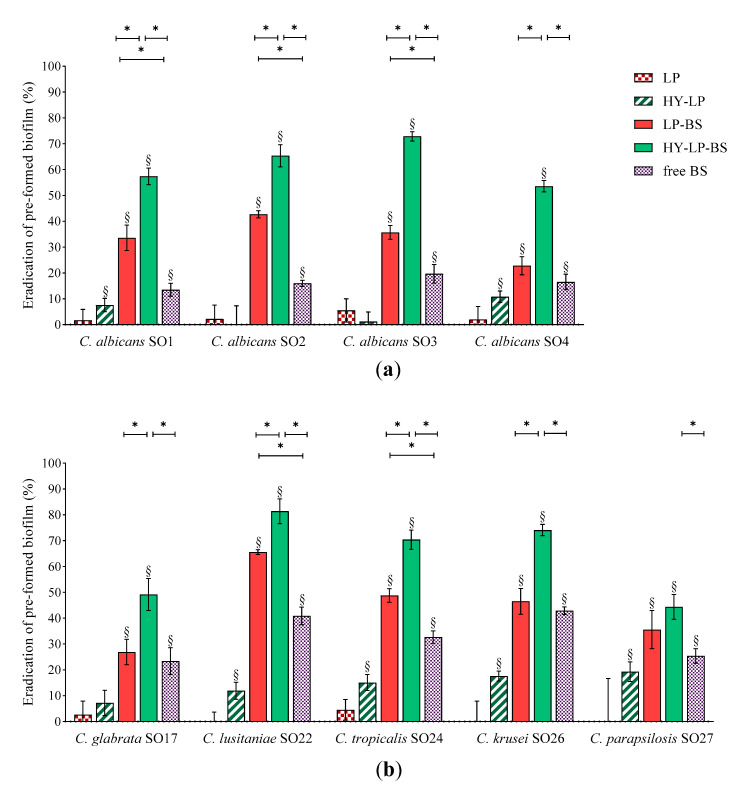
Anti-biofilm activity: dispersal of pre-formed (**a**) *C. albicans* and (**b**) non-*albicans* biofilms. Results are expressed as eradication percentage (mean ± SD, *n* = 5). The statistical significance with respect to untreated control was reported, §: *p* < 0.01. Statistical differences between BS, LP-BS and HY-LP-BS were also calculated, *: *p* < 0.05.

*Candida* biofilms are extremely difficult to eradicate since they are five to eightfold more resistant to azole drugs compared to planktonic cells. This behavior depends on several factors, such as high concentration of fungal cells inside biofilms and the presence of an extracellular matrix, which could provide evasion from host immunity and limited diffusion of antifungal agents inside the biofilm [49]. Thus, we sought for the ability of free BS, LP-BS and HY-LP-BS to eradicate already established biofilm. Biofilms of *Candida* were formed for 72 h and then adherent cells were treated for 48 h with free BC1-BS or phospholipid vesicles. Results are expressed as eradication percentages with respect to untreated control and depicted in Figure 4.

Free BS induced significant biofilm dispersal on all the *Candida* strains tested, indicating that the employment of a biosurfactant can be a promising strategy not only for the prevention but also for the treatment of an ongoing *Candida* infection. However, compared to the inhibition of biofilm formation, free BS was less effective in eradication assays, with eradication rates ranging from 13% to 43%. This can be attributed to the difficulty BS has in penetrating the biofilm matrix. In this regard, the formulation of BS in lipid nanocarriers may improve its availability at the site of action. Indeed, LP-BS significantly increased biosurfactant ability to eradicate the biofilm of three *C. albicans* strains out of four, probably as a consequence of a better penetration of lipophilic vesicles into the extracellular matrix of the biofilm [17]. This result is in agreement with another study reporting that the delivery of an azole drug by means of liposomes improved the eradication of pre-formed *C. albicans* biofilm [16].

The impact of LP-BS on non-*albicans* species seemed to be less marked, since conventional liposomes were able to significantly improve the eradication effects of BS only towards *C. lusitaniae* SO22 and *C. tropicalis* SO24 biofilms. Although hyaluronic acid is reported to be a component of extracellular matrix [50], some authors demonstrated the anti-biofilm activity of this polymer against bacteria such as *Streptococcus epidermidis*, *S. aureus* and *Escherichia coli* [51,52,53]. The capability of HY-LP to weakly disturb the biofilm of six *Candida* strains (eradication of ~10%), suggested that the employment of hyaluronic acid can be a valid strategy also to deal with fungal biofilm. Notably, HY-LP-BS revealed higher dispersal performance against all *Candida* strains tested as compared to free BS (*p* < 0.05), with eradication rates of 53–72% for *C. albicans* strains (Figure 4a and 44–81% for non-*albicans* strains (Figure 4b). This finding is also coherent with what was previously observed in inhibition assays, and confirms that HY-LP-BS displayed the maximal overall effect on *Candida* biofilm.

## 3. Materials and Methods

### 3.1. Materials

Phospholipon 90G from soybean lecithin (containing not less than 94% phosphatidylcholine) and sodium hyaluronate (HY; MW: 800–1200 kDa) were provided from Lipoid GmbH (Ludwigshafen, Germany) and Farmalabor (Canosa di Puglia, Italy), respectively. Mucin (type II: crude, from porcine stomach), crystal violet, L-cysteine hydrochloride monohydrate and all solvents were from Sigma-Aldrich (Milan, Italy).

De Man, Rogosa and Sharpe medium (MRS) was supplied by Difco (Detroit, MI, USA) and Sabouraud dextrose medium (SD) from Oxoid (Basingstoke, UK). GasPak EZ was purchased from Becton Dickinson and Company (Sparks, MD, USA).

Ultrapure water (18.2 MΏ cm) was obtained by means of a MilliQ apparatus by Millipore (Milford, MA, USA). The phosphate buffer (PBS) was prepared with the following composition: 2.38 g/L Na_2_HPO_4_, 0.19 g/L KH_2_PO_4_ and 8 g/L NaCl, pH = 7.4.

### 3.2. Microorganisms and Culture Conditions

*L. crispatus* BC1 isolation was done from the vaginal swab of a healthy premenopausal woman and the protocol was approved by the Ethics Committee of the University of Bologna, Italy (52/2014/U/Tess) [13]. BC1 strain culture medium was composed of 55 g/L *w/v* of MRS powder and 0.05% *w/v* L-cysteine; lactobacilli were grown at 37 °C for 24 h in anaerobic jars containing GasPak EZ.

Nine clinical isolates of *Candida* spp. were employed in the present study, namely *C. albicans* SO11-SO4, *C. glabrata* SO17, *C. lusitaniae* SO22, *C. tropicalis* SO24, *C. krusei* SO26 and *C. parapsilosis* SO27. They all belong to a collection of yeasts isolated from vaginal swabs of premenopausal, VVC affected women during routine diagnostic procedures at the Microbiology Laboratory of Sant’Orsola-Malpighi University Hospital of Bologna, Italy. *Candida* isolates were grown in SD medium aerobically at 35 °C for 24–48 h [9].

### 3.3. Isolation of BS from L. crispatus BC1

BS was isolated from *L. crispatus* BC1 cell surface as previously reported [9]. Briefly, BC1 was grown in 1 L of MRS broth for 48 h in anaerobic conditions, then microbial suspension was centrifuged at 10,000× *g* for 15 min. The cell pellet was washed twice in sterile distilled water, resuspended in 240 mL of sterile PBS and gently stirred at room temperature for 2 h to release the cell-bound BS. The sample was then centrifuged and the supernatant filtered through a 0.22 μm pore size filter (PES 0.22 μm syringe filters, VWR International, Milan, Italy). The obtained solution was subjected to dialysis against demineralized water in a Cellu-Sep^©^ membrane (molecular weight cut-off 6000–8000 Da; Spectra/Por 2 dialysis membrane, Spectrum Laboratories Inc., Rancho Dominguez, CA, USA) for 24 h at room temperature, and freeze-dried at 0.01 atm and −45 °C (Christ Freeze Dryer ALPHA 1–2, Milan, Italy). About 60 mg of lyophilized BS was obtained from 1 L of *L. crispatus* BC1 culture. As reported by De Gregorio et al. [9], BS produced by *L. crispatus* BC1 possessed a lipopeptidic structure and a critical micelle concentration of 2 mg/mL, evaluated by Fourier-transform infrared spectroscopy and mass spectrometric analysis and by the ring method using a tensiometer equipped with a platinum ring, respectively.

### 3.4. Preparation of Liposomes and Hyalurosomes

For the preparation of liposomes (LP) and hyalurosomes (HY-LP), the previously reported film rehydration and extrusion method was employed with some modifications [22,54]. Specifically, L-α-phosphatidylcholine (30 mg/mL) was dissolved in a mixture of CHCl_3_-CH_3_OH (2.5 mL, 9:1 *v/v*). The organic phase was evaporated in a round-bottomed flask by using a rotatory evaporator (Buchi Rotavapor R-200, Flawil, Switzerland) under reduced pressure (80 mbar) at 55 °C, 210 rpm for 120 min. At the end of this period, a dry lipid film was obtained and hydrated by using the rotatory evaporator (T = 25 °C, 210 rpm) with 10 mL of saline solution (NaCl 0.9% *w/v*) for 1 h. HY-LP were prepared through rehydration of the lipid film with a sodium hyaluronate solution, obtained by dissolving the polymer in saline solution (0.1 mg/mL) for 30 min under stirring at 200 rpm. For the preparation of LP and HY-LP containing BC1-BS, the biosurfactant was dissolved in the saline solution (1.25 mg/mL) without or with hyaluronic acid. To reduce and homogenize vesicle size, all the suspensions were extruded 10 times through a polycarbonate membrane with a pore size of 200 nm (LiposoFast manual syringe extruder, Avestin Europe GmbH, Mannheim, Germany).

The prepared suspensions were named as follows: LP and LP-BS for liposomes, without or with BC1-BS, respectively; HY-LP and HY-LP-BS for hyalurosomes without or with BC1-BS, respectively.

### 3.5. Determination of Vesicle Size Distribution and Zeta Potential

For the determination of phospholipid vesicle size, PDI and ζ potential, suspensions were diluted (1:500; *v/v*) in ultrapure water (18.2 MΏ cm, MilliQ apparatus by Millipore, Milford, MA, USA). Size and PDI were obtained through photon-correlation spectroscopy (PCS) using a Brookhaven 90-PLUS instrument (Brookhaven Instruments Corp., Holtsville, NY, USA) with He-Ne laser beam at a wavelength of 532 nm (scattering angle of 90°). A Malvern Zetasizer 3000 HS instrument (Malvern Panalytical Ltd., Malvern, UK) was used for ζ potential measurement. All the experiments were performed in triplicate.

### 3.6. Vesicle Physical Stability

The physical stability of the prepared phospholipid vesicles was evaluated over a period of storage of 180 days at 4–8 °C. At determined time intervals (2, 6, 12, 30, 60 and 180 days), aliquots of vesicle suspensions were diluted in ultrapure water as described in Section 3.5, and changes in vesicle size and PDI were monitored using PCS. All the experiments were performed in triplicate.

### 3.7. Mucoadhesive Properties

Mucoadhesive properties were investigated by measuring the turbidity of the suspensions containing phospholipid vesicles together with mucin [16,39,40,55]. Mucin was dispersed in water (0.08% *w/v*) for 6 h. Subsequently, the mucin dispersion was centrifuged at 7500 rpm (GS-15R Centrifuge, Beckman Coulter, Milan, Italy) for 20 min. The supernatant was isolated, mixed with the different vesicle suspensions (1:1 *v/v*) and vortexed for 1 min. After 3 h, samples were opportunely diluted in water and the turbidity was immediately measured at 650 nm through UV–Visible Spectrophotometer (Shimadzu Corporation, Sydney, Australia). The absorbance (ABS) of mucin dispersion itself and vesicle suspension without mucin were also recorded as controls. All the experiments were performed in triplicate.

### 3.8. Evaluation of Anti-Biofilm Activity of Free BS, Liposomes and Hyalurosomes

The anti-biofilm potential of free BS, LP-BS and HY-LP-BS was assessed by considering two different mechanisms of action: the inhibition of biofilm formation and the dispersal of pre-formed biofilm [16].

*Candida* suspensions were prepared in SD medium at a final concentration of 10^6^ CFU/mL and used as starting inoculum for both assays. BS was solubilized in sterile saline at 1.25 mg/mL as in the liposomal formulations and filtered through a 0.22 μm pore size filter.

#### 3.8.1. Inhibition of the Biofilm Development

For the inhibition assay, sterile 96 multi-well flat-bottomed plates (Corning Inc., Pisa, Italy) were filled with 100 μL of *Candida* suspension and 100 μL of BS in the following forms: (i) free BS, (ii) LP-BS and (iii) HY-LP-BS. Plain liposomes (LP) and plain hyalurosomes (HY-LP) were also tested for comparison. Controls contained 100 μL of fungal suspension and 100 μL of PBS. Wells filled with SD only served as blank, while wells containing SD and phospholipid vesicles were included as sterility controls.

The multi-well plates were incubated at 35 °C with shaking (100 rpm) for 72 h to allow biofilm development. Afterwards, the liquid culture was removed and biofilm formation was quantified through crystal violet staining. Briefly, adherent cells were washed twice with 200 μL of PBS, fixed with 200 μL of absolute ethanol for 5 min and stained with 2% crystal violet (*w/v*) in 12% ethanol for 5 min. After washing the wells with PBS for three times to remove the excess stain, the dye bound to adherent yeasts was resolubilized with 200 μL of ethanol and the absorbance was measured at 595 nm (ABS 595) (EnSpire Multimode Plate Reader, PerkinElmer Inc., Waltham, MA, USA).

The inhibition of biofilm formation was expressed in percentage relative to the untreated control wells, following the equation reported (Equation (1)):

Inhibition of biofilm formation/biofilm eradication (%) = [1 − (mean ABS 595 sample/ mean ABS 595 control)] × 100(1)

#### 3.8.2. Biofilm Dispersal

For the eradication assay, the 96 multi-well plates were inoculated with 200 μL of *Candida* suspensions or SD medium only. After 72 h of incubation (35 °C, 100 rpm) the liquid cultures were removed, leaving only adherent biofilm in the wells. Biofilms were then treated with 100 μL of SD and 100 μL of free BS, LP-BS or HY-LP-BS. Control wells were supplied with SD only. LP and HY-LP were tested for comparison; blank and sterility controls were also included. Plates were further incubated at 35 °C with shaking (100 rpm) for 48 h and biofilm quantification was performed through the protocol described above. The biofilm eradication was expressed in percentage relative to the untreated control wells, as reported above (Equation (1)).

### 3.9. Statistical Analysis

All results were expressed as mean ± standard deviation (SD). Student’s *t*-test was applied for the comparison of two means, one-way ANOVA followed by Bonferroni correction was used for multiple comparison. GraphPad Prims version 9.0.0 for Windows (GraphPad Software, San Diego, CA, USA, www.graphpad.com) was employed for statistical analyses and differences were deemed significant for *p* < 0.05.

## 4. Conclusions

In the present work a biosurfactant from the vaginal strain *L. crispatus* BC1 was indagated as an alternative natural substance to counteract biofilms formed by clinically relevant *Candida* spp., which are often associated with both drug-resistance and recurrent outcomes. The BS was found to be active towards *C. albicans* and non-*albicans* strains, especially in inhibiting the formation of fungal biofilms. In order to favor the vaginal delivery and optimize its biological activity, two different lipidic nanocarriers, liposomes and hyalurosomes, containing the BS were successfully developed. LP-BS and HY-LP-BS revealed optimal sizes to target the vaginal mucosa and good ability to bind mucin, which is an important requisite to assure prolonged permanence of a locally delivered formulation in the site of action. Moreover, they were stable over the storage period of 180 days. Notably, the inclusion of BS inside liposomal formulations allowed enhancing of its anti-biofilm activity. In particular, HY-LP-BS revealed the best profile both in inhibiting *Candida* biofilms’ formation and in dispersing pre-formed biofilms. Although other studies are required to deeply investigate the potential employment of such formulations in humans, the evaluation of anti-biofilm properties represents a first step in the research and development of new biotherapeutic approaches to counteract VVC. Our preliminary results underline that BS-containing liposomes can be a promising strategy to contain vulvovaginal *Candida* infections.

## Figures and Tables

**Table 1:** Size (nm), particle size distribution (PDI) and ζ potential (mV) of the different phospholipid vesicles.

Phospholipid Vesicle Codename	Size (nm)	PDI	ζ Potential (mV)
LP	310 ± 3	0.29 ± 0.02	−30.86 ± 0.76
HY-LP	379 ± 7	0.30 ± 0.01	−29.33 ± 1.17
LP-BS	284 ± 6	0.31 ± 0.01	−23.04 ± 0.91
HY-LP-BS	342 ± 12	0.29 ± 0.04	−22.34 ± 0.13

## Data Availability

Data is contained within the article.

## References

[1] Lohse M., Gulati M., Johnson A., Nobile C. (2018). Development and regulation of single- and multi-species *Candida albicans* biofilm. Nat. Rev. Microbiol..

[2] Dominguez E., Zarnowski R., Sanchez H., Covelli A.S., Westler W.M., Azadi P., Nett J., Mitchell A.P., Andes D.R. (2018). Conservation and Divergence in the *Candida* Species Biofilm Matrix Mannan-Glucan Complex Structure, Function, and Genetic Control. MBio.

[3] Denning D., Kneale M., Sobel J., Rautemaa-Richardson R. (2018). Global burden of recurrent vulvovaginal candidiasis: A systematic review. Lancet Infect. Dis..

[4] Willems H., Ahmed S., Liu J., Xu Z., Peters B. (2020). Vulvovaginal Candidiasis: A Current Understanding and Burning Questions. J. Fungi.

[5] Anestopoulos I., Kiousi D.E., Klavaris A., Galanis A., Salek K., Euston S.R., Pappa A., Panayiotidis M. (2020). Surface Active Agents and Their Health-Promoting Properties: Molecules of Multifunctional Significance. Pharmaceutics.

[6] Gudiña E.J., Rangarajan V., Sen R., Rodrigues L.R. (2013). Potential therapeutic applications of biosurfactants. Trends Pharmacol. Sci..

[7] Sharma D., Singh Saharan B. (2014). Simultaneous Production of Biosurfactants and Bacteriocins by Probiotic *Lactobacillus casei* MRTL3. Int. J. Microbiol..

[8] Zakaria Gomaa E. (2013). Antimicrobial and anti-adhesive properties of biosurfactant produced by lactobacilli isolates, biofilm formation and aggregation ability. J. Gen. Appl. Microbiol..

[9] De Gregorio P.R., Parolin C., Abruzzo A., Luppi B., Protti M., Mercolini L., Silva J.A., Giordani B., Marangoni A., Nader-Macías M.E.F. (2020). Biosurfactant from vaginal *Lactobacillus crispatus* BC1 as a promising agent to interfere with *Candida* adhesion. Microb Cell Fact..

[10] Parolin C., Frisco G., Foschi C., Giordani B., Salvo M., Vitali B., Marangoni A., Calonghi N. (2018). *Lactobacillus crispatus* BC5 Interferes With *Chlamydia trachomatis* Infectivity Through Integrin Modulation in Cervical Cells. Front. Microbiol..

[11] Ceccarani C., Foschi C., Parolin C., D’Antuono A., Gaspari V., Consolandi C., Laghi L., Camboni T., Vitali B., Severgnini M. (2019). Diversity of vaginal microbiome and metabolome during genital infections. Sci. Rep..

[12] Ñahui Palomino R.A., Vanpouille C., Laghi L., Parolin C., Melikov K., Backlund P., Vitali B., Margolis L. (2019). Extracellular vesicles from symbiotic vaginal lactobacilli inhibit HIV-1 infection of human tissues. Nat. Commun..

[13] Parolin C., Marangoni A., Laghi L., Foschi C., Ñahui Palomino R.A., Calonghi N., Cevenini R., Vitali B. (2015). Isolation of Vaginal Lactobacilli and Characterization of Anti-*Candida* Activity. PLoS ONE.

[14] Vitali B., Abruzzo A., Parolin C., Ñahui Palomino R.A., Dalena F., Bigucci F., Cerchiara T., Luppi B. (2016). Association of *Lactobacillus crispatus* with fructo-oligosaccharides and ascorbic acid in hydroxypropyl methylcellulose vaginal insert. Carbohydr. Polym..

[15] Giordani B., Basnet P., Mishchenko E., Luppi B., Škalko-Basnet N. (2019). Utilizing Liposomal Quercetin and Gallic Acid in Localized Treatment of Vaginal *Candida* Infections. Pharmaceutics.

[16] Abruzzo A., Giordani B., Parolin C., Vitali B., Protti M., Mercolini L., Cappelletti M., Fedi S., Bigucci F., Cerchiara T. (2018). Novel mixed vesicles containing lactobacilli biosurfactant for vaginal delivery of an anti-*Candida* agent. Eur. J. Pharm. Sci..

[17] Rukavina Z., Vani Ž. (2016). Current trends in development of liposomes for targeting bacterial biofilms. Pharmaceutics.

[18] Giordani B., Costantini P.E., Fedi S., Cappelletti M., Abruzzo A., Parolin C., Foschi C., Frisco G., Calonghi N., Cerchiara T. (2019). Liposomes containing biosurfactants isolated from *Lactobacillus gasseri* exert antibiofilm activity against methicillin resistant *Staphylococcus aureus* strains. Eur. J. Pharm. Biopharm..

[19] Vicario-de-la-Torre M., Caballo-González M., Vico E., Morales-Fernández L., Arriola-Villalobos P., De Las Heras B., Benítez-Del-Castillo J.M., Guzmán M., Millar T., Herrero-Vanrell R. (2018). Novel Nano-Liposome Formulation for Dry Eyes with Components Similar to the Preocular Tear Film. Polymers.

[20] Bohbot J.M., de Belilovsky C., Brami G., Mares P. (2015). Efficacy of a medical device containing liposomal hyaluronic acid against vulvo-vaginal dryness. Gynecol. Obs. Fertil..

[21] Mowbray C.A., Shams S., Chung G., Stanton A., Aldridge P., Suchenko A., Pickard R.S., Ali A.S., Hall J. (2018). High molecular weight hyaluronic acid: A two-pronged protectant against infection of the urogenital tract?. Clin. Transl. Immunol..

[22] Abruzzo A., Cappadone C., Farruggia G., Luppi B., Bigucci F., Cerchiara T. (2020). Glycyrrhetinic Acid Liposomes and Hyalurosomes on Spanish Broom, Flax, and Hemp Dressings to Heal Skin Wounds. Molecules.

[23] Castangia I., Caddeo C., Manca M.L., Casu L., Latorre A.C., Díez-Sales O., Ruiz-Saurí A., Bacchetta G., Fadda A.M., Manconi M. (2015). Delivery of liquorice extract by liposomes and hyalurosomes to protect the skin against oxidative stress injuries. Carbohydr. Polym..

[24] Duangjit S., Obata Y., Sano H., Kikuchi S., Onuki Y., Opanasopit P., Ngawhirunpat T., Maitani Y., Takayama K. (2012). Menthosomes, novel ultradeformable vesicles for transdermal drug delivery: Optimization and characterization. Biol. Pharm. Bull..

[25] Duangjit S., Pamornpathomkul B., Opanasopit P., Rojanarata T., Obata Y., Takayama K., Ngawhirunpat T. (2014). Role of the charge, carbon chain length, and content of surfactant on the skin penetration of meloxicam-loaded liposomes. Int. J. Nanomed..

[26] El Sayyad M.K., Zaky A.A., Samy A.M. (2017). Fabrication and characterization of sildenafil citrate loaded transfersomes as a carrier for transdermal drug delivery. Pharm. Pharmacol. Int. J..

[27] Bnyan R., Khan I., Ehtezazi T., Saleem I., Gordon S., O’Neill F., Roberts M. (2018). Surfactant Effects on Lipid-Based Vesicles Properties. J. Pharm. Sci..

[28] Das Neves J., Bahia M.F., Amiji M.M., Sarmento B. (2011). Mucoadhesive nanomedicines: Characterization and modulation of mucoadhesion at the nanoscale. Expert Opin. Drug Deliv..

[29] Verma D.D., Verma S., Blume G., Fahr A. (2003). Particle size of liposomes influences dermal delivery of substances into skin. Int. J. Pharm..

[30] Danaei M., Dehghankhold M., Ataei S., Davarani F.H., Javanmard R., Dokhani A., Khorasani S., Mozafari M.R. (2018). Impact of Particle Size and Polydispersity Index on the Clinical Applications of Lipidic Nanocarrier Systems. Pharmaceutics.

[31] Chen M., Liu X., Fahr A. (2011). Skin penetration and deposition of carboxyfluorescein and temoporfin from different lipid vesicular systems: In vitro study with finite and infinite dosage application. Int. J. Pharm..

[32] Castangia I., Manca M.L., Catalán-Latorre A., Maccioni A.M., Fadda A.M., Manconi M. (2016). Phycocyanin-encapsulating hyalurosomes as carrier for skin delivery and protection from oxidative stress damage. J. Mater. Sci. Mater. Med..

[33] Kassem M.A., Aboul-Einien M.H., El Taweel M.M. (2018). Dry Gel Containing Optimized Felodipine-Loaded Transferosomes: A Promising Transdermal Delivery System to Enhance Drug Bioavailability. AAPS PharmSciTech.

[34] Mir-Palomo S., Nácher A., Ofelia Vila-Busó M.A., Caddeo C., Manca M.L., Saurí A.R., Escribano-Ferrer E., Manconi M., Díez-Sales O. (2020). Co-loading of finasteride and baicalin in phospholipid vesicles tailored for the treatment of hair disorders. Nanoscale.

[35] Maritim S., Boulas P., Lin Y. (2020). Comprehensive analysis of liposome formulation parameters and their influence on encapsulation, stability and drug release in glibenclamide liposomes. Int. J. Pharm..

[36] Perttu E.K., Kohli A.G., Szoka F.C. (2012). Inverse-phosphocholine lipids: A remix of a common phospholipid. J. Am. Chem. Soc..

[37] Scherer P.G., Seelig J. (1989). Electric charge effects on phospholipid headgroups. Phosphatidylcholine in mixtures with cationic and anionic amphiphiles. Biochemistry.

[38] Vanić Ž., Škalko-Basnet N. (2013). Nanopharmaceuticals for improved topical vaginal therapy: Can they deliver?. Eur. J. Pharm. Sci..

[39] Sallam M.A., Helal H.M., Mortada S.M. (2016). Rationally designed nanocarriers for intranasal therapy of allergic rhinitis: Influence of carrier type on in vivo nasal deposition. Int. J. Nanomed..

[40] Ungaro F., d’Angelo I., Coletta C., d’Emmanuele di Villa Bianca R., Sorrentino R., Perfetto B., Tufano M.A., Miro A., La Rotonda M.I., Quaglia F. (2012). Dry powders based on PLGA nanoparticles for pulmonary delivery of antibiotics: Modulation of encapsulation efficiency, release rate and lung deposition pattern by hydrophilic polymers. J. Control. Release.

[41] Sosnik A., das Neves J., Sarmento B. (2014). Mucoadhesive polymers in the design of nano-drug delivery systems for administration by non-parenteral routes: A review. Prog. Polym. Sci..

[42] Gulati M., Nobile C.J. (2016). *Candida albicans* biofilms: Development, regulation, and molecular mechanisms. Microbes Infect..

[43] Rodríguez-Cerdeira C., Gregorio M.C., Molares-Vila A., López-Barcenas A., Fabbrocini G., Bardhi B., Sinani A., Sánchez-Blanco E., Arenas-Guzmán R., Hernandez-Castro R. (2019). Biofilms and vulvovaginal candidiasis. Colloids Surf. B Biointerfaces.

[44] de Araujo L.V., Guimarães C.R., da SilvaMarquita R.L., Santiago V.M.J., de Souza M.P., Nitschke M., Guimarães Freire D.M. (2016). Rhamnolipid and surfactin: Anti-adhesion/antibiofilm and antimicrobial effects. Food Control..

[45] Paraszkiewicz K., Moryl M., Płaza G., Bhagat D., Satpute S.K., Bernat P. (2019). Surfactants of microbial origin as antibiofilm agents. Int. J. Environ. Heal. Res..

[46] Itapary Dos Santos C., Ramos França Y., Duarte Lima Campos C., Quaresma Bomfim M.R., Oliveira Melo B., Assunção Holanda R., Santos V.L., Gomes Monteiro S., Buozzi Moffa E., Souza Monteiro A. (2019). Antifungal and antivirulence activity of vaginal *Lactobacillus* spp. products against *Candida* vaginal isolates. Pathogens.

[47] Sakai A., Akifusa S., Itano N., Kimata K., Kawamura T., Koseki T., Takehara T., Nishihara T. (2007). Potential role of high molecular weight hyaluronan in the anti-*Candida* activity of human oral epithelial cells. Med. Mycol..

[48] Stevan M., Fusato E., Armanini D., Bertoloni G., De Seta F., Leli C., Rassu M. (2017). In vitro effects of glycyrrhetinic acid and hyaluronic acid on the growth of vulvovaginal *Candida albicans* and other yeasts. Microbiol. Med..

[49] Silva S., Rodrigues C.F., Araújo D., Rodrigues M.E., Henriques M. (2017). *Candida* species biofilms’ antifungal resistance. J. Fungi.

[50] Ardizzoni A., Neglia R.G., Baschieri M.C., Cermelli C., Caratozzolo M., Righi E., Palmieri B., Blasi E. (2011). Influence of hyaluronic acid on bacterial and fungal species, including clinically relevant opportunistic pathogens. J. Mater. Sci. Mater. Med..

[51] Drago L., Cappelletti L., De Vecchi E., Pignataro L., Torretta S., Mattina R. (2014). Antiadhesive and antibiofilm activity of hyaluronic acid against bacteria responsible for respiratory tract infections. APMIS.

[52] Junter G.A., Thébault P., Lebrun L. (2016). Polysaccharide-based antibiofilm surfaces. Acta Biomater..

[53] Romanò C.L., De Vecchi E., Bortolin M., Morelli I., Drago L. (2017). Hyaluronic acid and its composites as a local antimicrobial/antiadhesive barrier. J. Bone Jt. Infect..

[54] Uchino T., Lefeber F., Gooris G., Bouwstra J. (2011). Physicochemical characterization of drug-loaded rigid and elastic vesicles. Int. J. Pharm..

[55] Cerchiara T., Abruzzo A., di Cagno M., Bigucci F., Bauer-Brandl A., Parolin C., Vitali B., Gallucci M.C., Luppi B. (2015). Chitosan based micro- and nanoparticles for colon-targeted delivery of vancomycin prepared by alternative processing method. Eur. J. Pharm. Biopharm..

